# TADCompare: An R Package for Differential and Temporal Analysis of Topologically Associated Domains

**DOI:** 10.3389/fgene.2020.00158

**Published:** 2020-03-10

**Authors:** Kellen G. Cresswell, Mikhail G. Dozmorov

**Affiliations:** Department of Biostatistics, Virginia Commonwealth University, Richmond, VA, United States

**Keywords:** Hi-C, chromosome conformation capture, topologically associated domains (TADs), differential analysis, TADCompare

## Abstract

Recent research using chromatin conformation capture technologies, such as Hi-C, has demonstrated the importance of topologically associated domains (TADs) and smaller chromatin loops, collectively referred hereafter as “interacting domains.” Many such domains change during development or disease, and exhibit cell- and condition-specific differences. Quantification of the dynamic behavior of interacting domains will help to better understand genome regulation. Methods for comparing interacting domains between cells and conditions are highly limited. We developed TADCompare, a method for differential analysis of boundaries of interacting domains between two or more Hi-C datasets. TADCompare is based on a spectral clustering-derived measure called the eigenvector gap, which enables a loci-by-loci comparison of boundary differences. Using this measure, we introduce methods for identifying differential and consensus boundaries of interacting domains and tracking boundary changes over time. We further propose a novel framework for the systematic classification of boundary changes. Colocalization- and gene enrichment analysis of different types of boundary changes demonstrated distinct biological functionality associated with them. TADCompare is available on https://github.com/dozmorovlab/TADCompare and Bioconductor (submitted).

## 1. Introduction

Recent research indisputably proves the importance of the three-dimensional (3D) genome organization in regulating gene expression and other genomic processes (Osborne et al., 2004; Schoenfelder et al., 2010a,b; Tanizawa et al., 2010; Steensel, 2011; Li et al., 2012; Papantonis and Cook, 2013; Shavit and Lio, 2014; Symmons et al., 2014; Mifsud et al., 2015; Sexton and Cavalli, 2015; Franke et al., 2016; Mora et al., 2016). The 3D genomic structures consists of chromosome territories (Cremer and Cremer, 2010), A/B compartments corresponding to active/repressed chromatin (Lieberman-Aiden et al., 2009; Rao et al., 2014), topologically associated domains (TADs) (Jackson and Pombo, 1998; Ma et al., 1998; Dekker et al., 2002; Dixon et al., 2012; Nora et al., 2012; Sexton et al., 2012; Bonev et al., 2017), smaller sub-TADs (Phillips-Cremins and Corces, 2013; Rao et al., 2014) and chromatin loops (Dowen et al., 2014; Rao et al., 2014; Denker and Laat, 2016; Ji et al., 2016). These structures help to regulate global gene expression (de Laat and Grosveld, 2003; Osborne et al., 2004; Schoenfelder et al., 2010a,b; Tanizawa et al., 2010; Steensel, 2011; Li et al., 2012; Papantonis and Cook, 2013; Shavit and Lio, 2014; Symmons et al., 2014; Mifsud et al., 2015; Sexton and Cavalli, 2015; Franke et al., 2016; Mora et al., 2016). Consequently, coordinated changes in the 3D structures (Yaffe and Tanay, 2011; Dai and Dai, 2012; Symmons et al., 2014) determine cell type-specific gene expression and identity (Schoenfelder et al., 2010b; Dekker et al., 2013; Jin et al., 2013; Phillips-Cremins and Corces, 2013; Dowen et al., 2014; Rao et al., 2014; Vietri Rudan et al., 2015; Ji et al., 2016), guide recombination (Jhunjhunwala et al., 2009), X chromosome inactivation (Nora et al., 2012; Crane et al., 2015). Many 3D structures are largely invariant between different cell types, and even conserved between mammalian species (Dixon et al., 2012; Nora et al., 2012; Naumova et al., 2013; Pope et al., 2014; Rao et al., 2014; Vietri Rudan et al., 2015), indicating their high biological importance during genome evolution.

Despite the high level of conservation, recent research uncovered the dynamic nature of the 3D genomic structures, and this plasticity accompanies various biological functions and phenomena (Yu and Ren, 2017). In Drosophila, exposure to heat-shock caused local changes in certain TAD boundaries resulting in TAD merging (Li et al., 2015). Another recent study showed that during motor neuron (MN) differentiation in mammals, TAD and sub-TAD boundaries in the Hox cluster are not rigid, and their plasticity is linked to changes in gene expression during differentiation (Narendra et al., 2016). The global organization of the 3D genomic structure is found in mitosis (Nagano et al., 2017), the earliest stages of mammalian lineage development (Dixon et al., 2015; Bonev et al., 2017; Du et al., 2017; Ke et al., 2017), and somatic cell reprogramming of pluripotent stem cells (Novo et al., 2018; Zhang et al., 2018). Fusion of TADs (Nora et al., 2012; Dowen et al., 2014; Guo et al., 2015; Sanborn et al., 2015; Tang et al., 2015; Flavahan et al., 2016; Fudenberg et al., 2016), creation or destruction of sub-TADs within existing TAD boundaries (Lupiáñez et al., 2016; Taberlay et al., 2016), and/or switching TAD states between active and inactive conformations (Lieberman-Aiden et al., 2009; Dixon et al., 2012) has been associated with a variety of phenotypes (Misteli, 2010; Krijger and Laat, 2016; Spielmann et al., 2018), ranging from limb malformation (Lupiáñez et al., 2016), congenital disorders (Ibn-Salem et al., 2014), to cancer (Mitelman, 2000; Rickman et al., 2012; Gr‘`oschel et al., 2014; Barutcu et al., 2015; Corces and Corces, 2016; Flavahan et al., 2016; Hnisz et al., 2016; Krijger and Laat, 2016; Lupiáñez et al., 2016; Valton and Dekker, 2016). Chromatin loops are even more dynamic and change during the cell cycle and other cellular events (Sanborn et al., 2015; Fudenberg et al., 2016; Golfier et al., 2019). These observations highlight the importance of studying changes in the boundaries of interacting domains as a means to understand genomic regulation. However, methods for identifying these changes remain underdeveloped.

To our knowledge, there are only three methods that can be adapted for detecting changes in boundaries of interacting domains; the majority have been developed for the detection of TAD-specific boundary changes. Among the three methods, localtadsim (Sauerwald et al., 2020), HiCDB (Chen et al., 2018), and DiffTAD (Zaborowski and Wilczynski, 2016), none provide an intuitive, easy to use way of calling differential boundaries. Both localtadsim and DiffTAD are two-step procedures requiring separately defined TADs and comparing them using a command-line utility. HiCDB has a built-in TAD caller but does not allow for comparisons of chromosome-specific contact matrices. All three methods require highly specific data types and file names to be able to run. The lack of usability is compounded with issues, such as a lack of upkeep, slow runtimes, and lack of statistical rigor (Supplementary Methods).

As the costs of Hi-C data continue to drop, several studies started to investigate the dynamics of 3D changes over time. The most notable applications include cell differentiation studies (Bonev et al., 2017), embryonic development (Du et al., 2017; Hug et al., 2017; Ke et al., 2017), cancer progression (Zhou et al., 2019). Typically, TAD boundary changes over time are quantified by overlap (Du et al., 2017; Hug et al., 2017) and classified into distinct patterns (Zhou et al., 2019). However, general-purpose methods for systematic analysis of boundary changes over time do not exist.

The number of replicates for Hi-C experiments continue to rise, requiring methods for defining consistent boundaries of interacting domains across replicates of Hi-C data. Two primary approaches have been developed to identify TAD boundaries across multiple replicates. The first approach involves merging all replicates into a consensus contact matrix and then calling interacting domains [e.g., Arrowhead (Rao et al., 2014)]. The second is to call domains on individual replicates and aggregate them. A third approach available in the TADBit tool (Serra et al., 2017) allows for the alignment of TAD boundaries to a reference set of boundaries. This method relies on the reference set being “true boundaries” and is potentially sensitive to the selection of reference boundaries. Altogether, methods for detecting consensus boundaries of interacting domains across Hi-C datasets remain underdeveloped.

We developed TADCompare, an R package aimed at providing a fast, accurate, user-friendly, and well-documented approach to differential analysis of boundaries of TADs and chromatin loops. We introduce a method based on the boundary score statistic (Cresswell et al., 2019) and use it to identify five types of boundary changes. The method is extended to allow for calling consensus boundaries and comparing them between groups of Hi-C replicates. We further demonstrate how the boundary score statistic may be used to analyze the dynamics of boundaries of interacting domains over the time course. For both differential boundary detection and time course analysis, we provide novel terminology for the classification of boundary changes. We demonstrated the robustness of TADCompare using simulated data with pre-defined interacting domains (Forcato et al., 2017) and its ability to reveal distinct biological roles of different boundary changes. In summary, TADCompare provides an all-in-one pipeline from consensus boundary calling to differential boundary detection, including time course. The output is formatted in a commonly used BED format that allows for flexible downstream analyses and visualization.

## 2. Methods

### 2.1. Representation of Hi-C Data as a Graph

For a given Hi-C experiment, Hi-C data is represented by a chromosome-specific contact matrix *C* of non-overlapping regions (aka bins) of size *r* (resolution of the data). Each entry *C*_*ij*_ corresponds to the number of contacts between region *i* and region *j*. Previous work has shown that this contact matrix is essentially an analog of the adjacency matrix found in graph theory and Hi-C data can be thought of as a naturally occurring graph where edges are contacts and vertices are genomic regions (Boulos et al., 2013; Wang et al., 2013, 2019; Cresswell et al., 2019), or genes associated with them (Merelli et al., 2013). The graph representation of Hi-C data is the foundation of our method and allows us to use a graph-clustering based approach to identify and analyze TADs.

### 2.2. Calculating the Graph Spectrum

The first step of our method is to calculate the graph spectrum, defined as the eigenvectors of the Laplacian of an adjacency matrix. Using the interpretation of the contact matrix as a naturally occurring adjacency matrix, we calculate the Laplacian directly from the contact data. Briefly, the graph spectrum for a given contact matrix is calculated as follows:

1. Calculate the normalized Laplacian $\bar{L}$:
  $$\begin{array}{l} \bar{L}=D^{-\frac{1}{2}}CD^{-\frac{1}{2}} \end{array}$$
  where *D* = *diag*(**1**^*T*^*C*), where **1** is a column vector of size *C* where each entry is 1. *D* can be thought of as a vector containing the sum of the degrees for a given node.
2. Perform an eigendecomposition of the Laplacian:
  $$\begin{array}{l} \bar{L}v=\lambda v \end{array}$$
  In practice, we calculate the first two eigenvectors with the largest absolute values of eigenvalues and organize them into a matrix $\bar{V}$ with dimensions *i* × 2, where *i* is the number of regions in the contact matrix. $\bar{V}$ is referred to as the graph spectrum of the contact matrix.

### 2.3. Eigenvector Gap as a Measure of Pattern Change

**Figure 1:**
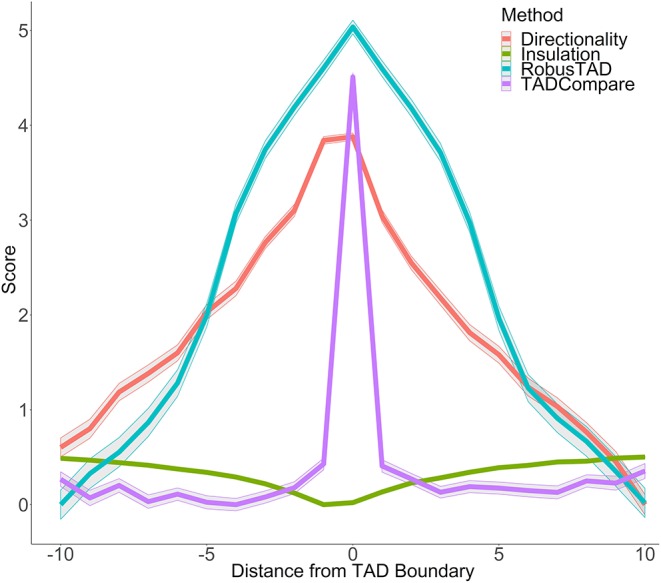
Boundary score distinguishes boundaries better than monotonic metrics. Boundary scores calculated with four methods: directionality index, insulation score, RobusTAD, and TADCompare are shown. X-axis—distance from the boundary, measured in bins (40 kb each), Y-axis—score (signed log10 values centered at zero). Results from five simulated contact matrices, 40 kb resolution, with manually annotated boundaries (Forcato et al., 2017) are shown.

We can think of each row of the matrix $\bar{V}$ as a quantification of the pattern of contacts in each region of the contact matrix. Previous work (Cresswell et al., 2019) has demonstrated that by taking the Euclidean distance between row *V*_*i*._ and its neighboring row *V*_(*i*+1)._, one can measure the similarity in the pattern of contacts between region *i* and region *i*+1 of the chromosome, termed “eigenvector gap.” A boundary between interacting regions manifests itself as a sudden break in the pattern of contacts. This pattern is reflected in the eigenvector gap by a spike in gap size followed by and preceded by smaller gaps (Figure 1). The eigenvector gap quantifies the degree of this break, acting as a proxy for TAD boundary likelihood. To calculate the eigenvector gaps, we perform the following procedure:

1. Normalize columns of $\bar{V}$ to sum to 1:
  $$\begin{array}{l} \begin{array}{c} {\hat{V}}_{ij}=\frac{{\bar{V}}_{ij}}{\left\|{\bar{V}}_{.j}\right\|} \end{array} \end{array}$$
  where the subscript. *j* corresponds to column *j*.
2. Normalize $\hat{V}$ and project onto a unit circle:
  $$\tilde{Z}=Diag(diag^{-\frac{1}{2}}({\hat{V}}_{i}{}_{.}{\hat{V}}_{i}{{}_{.}}^{T})){\hat{V}}_{i}{}_{.}$$
3. Calculate the distance between neighboring regions (rows *i* and *i*−1 of $\tilde{Z}$) and store in a vector *D*_*i*_:
  $$\begin{array}{l} D_{i}=\sqrt{{\left({\tilde{Z}}_{i1}-{\tilde{Z}}_{\left(i-1\right)1}\right)}^{2}+{\left({\tilde{Z}}_{i2}-{\tilde{Z}}_{\left(i-1\right)2}\right)}^{2}} \end{array}$$
  We refer to *D* as the vector where each entry *D*_*i*_ is referred to as an eigenvector gap. Formally, an eigenvector gap is the Euclidean distance between each successive row of the first two eigenvectors. In practical terms, the eigenvector gap for a given locus is a measure of how likely that loci is a boundary.

To maintain the association of each entry of the vector with its corresponding matrix region, a placeholder is used in the first entry of the vector. This is necessary because we cannot calculate an eigenvector gap for the first entry of the contact matrix due to a lack of left-bound neighbor. In mathematical terms, this means that for a matrix of size *n* the total number of eigenvector gaps is *n*−1.

### 2.4. Converting Eigenvector Gaps to Boundary Scores

We showed that the distribution of eigenvector gaps can be approximated by a log-normal distribution (Supplementary Figure 1). The log-normality allows us to convert the eigenvector gap values into boundary scores:

$$\begin{array}{l} B_{i}=\frac{\left(ln\left(D_{i}\right)-\mu \right)}{\sigma^{2}} \end{array}$$

where *ln*(*D*) ~ *N*(μ, σ^2^) where μ and σ^2^ are the mean and variance of the distribution of the natural log of the eigenvector gaps, respectively, and *B* is a vector of boundary scores with a N(0,1) distribution. In practice, this value is simply the Z-score for the natural log of eigenvector gaps.

### 2.5. Sliding Window Eigenvector Gap Calculation

The frequency of interactions decays following power law as the distance between the interacting regions increases (Lajoie et al., 2015). This decay leads to noisy and non-informative interactions farther off-diagonal of the contact matrix. To alleviate the effect of noisy distant interactions, we perform spectral decomposition within a fixed-size window that moves along the diagonal of the matrix. For instance, a window size of 15 bins (default setting, Supplementary Figure 2) means that only values within 15 bins of the diagonal will be used to calculate the eigenvector gap. The sliding window approach improves the performance of the eigenvector gap calculation (Cresswell et al., 2019). Additionally, it provides for faster calculations, operating on many small matrices instead of one large matrix. In general, we found that the results are robust to window size (Supplementary Figure 2). At higher levels of noise and sparsity, we found that larger windows tend to perform marginally better (Supplementary Figure 2). This is likely due to the fact that more data points are needed to capture pattern change in these scenarios. To achieve a good compromise on performance, we used a window size of 15 for each resolution.

### 2.6. Handling of Non-informative Bins

Non-informative bins refer to bins with <20% of non-zero interactions. This percentage is calculated based on regions within our sliding window. Such bins can introduce instability in the algorithm and lack important information. To counter this, we remove these bins before the analysis. This is done for both contact matrices such that, if one contact matrix contains a non-informative bin at a given location and the other does not, we remove it from both. This allows us to make a one-to-one comparison of bins.

### 2.7. Differential Analysis Using Boundary Scores

To define the differences between two contact matrices, *P* and *R*, we compare their eigenvector gaps *D*_*P*_ and *D*_*R*_, respectively. Given that $ln\left(D_{P}\right)\sim N\left(\mu_{P},\sigma_{P}^{2}\right)$ and $ln\left(D_{R}\right)\sim N\left(\mu_{R},\sigma_{R}^{2}\right)$, it follows that $ln\left(D_{P}\right)\;-\;ln\left(D_{R}\right)\sim N\left(\mu_{P}-\mu_{R},\sigma_{P}^{2}\;+\;\sigma_{R}^{2}\right)$. These results allow us to calculate a vector of differential boundary scores:

$$\begin{array}{l} DB_{i}=\frac{\left(ln\left(D_{Pi}\right)-ln\left(D_{Ri}\right)\right)-\left(\mu_{P}-\mu_{R}\right)}{\sigma_{P}^{2}+\sigma_{R}^{2}} \end{array}$$

or more simply,

$$\begin{array}{l} DB_{i}=\frac{\sigma_{P}^{2}B_{P}-\sigma_{R}^{2}B_{R}}{\sigma_{P}^{2}+\sigma_{R}^{2}} \end{array}$$

where *B*_*P*_ and *B*_*R*_ are the boundary scores for the *P* and *R* matrices, respectively. This score can be thought of as the difference in boundary likelihood for a given locus in two data sets. Due to the aforementioned normality of the difference in log eigenvector gaps, *DB*_*i*_ can be thought of as a simple z-score where *DB* ~ *N*(0, 1).

Boundary differences may be visualized using the package's TADcompare::DiffPlot function (Supplementary Figure 3C), or by external tools [e.g., HiCexplorer (Ramirez et al., 2018)].

### 2.8. Time Course Boundary Changes

Boundary scores provide a convenient method for modeling the change of boundaries over time. For a given boundary, or, any region of the genome, we can monitor the trajectory of the boundary score. Over time, we can define boundary score changes based on their deviation from a baseline level (typically, the first time point). It is expected that these scores will be relatively constant over time except in regions where a boundary appears or disappears. The trend across time points can be recorded and the pattern of change classified accordingly. Our implementation of time course boundary analysis allows for the usage of multiple replicates for a given time point. Briefly, at each region of the genome, the consensus boundary score is calculated, defined as the median of consensus scores across all replicates, and is then used to identify boundaries.

### 2.9. Gene Enrichment Testing

All gene enrichment testing was performed using the GREAT method (McLean et al., 2010) implemented in the rGREAT (Version 2.0) R package. Briefly, we detect genes within 5 kb upstream and 1 kb downstream of each type of boundary change, similar to the work of others (Chen et al., 2018). For each Gene Ontology (GO) and pathways, a hypergeometric test is then performed to determine the over-representation of boundary-associated genes. For all figures, we report results for GO Biological Processes. Results for GO Molecular Function, GO Cellular Component, MSigDB, and PANTHER pathways are reported in tables.

### 2.10. Colocalization Enrichment Testing

A permutation test was used to quantify the enrichment of colocalization of boundaries of interest with genomic annotations. Briefly, we flank each type of boundary change (differential or time course) by 50 kb on each side and calculate the mean number of genomic annotations across those regions (observed enrichment). Next, we generate two sets of bins, one the size of the boundaries which we are testing (considering the flanking) and another the size of all other bins. The difference in the mean number of genomic annotations colocalized with boundaries of interest was calculated for each set (expected enrichment). We repeat this procedure 10,000 times. We calculate the permutation *p*-value by taking the number of times the expected enrichment was greater than the observed enrichment, and dividing by 10000. α = 0.05 was set to assess statistical significance.

### 2.11. Data and Code Availability

All simulated data were downloaded from the HiCToolsCompare repository (Forcato et al., 2017). In total, we used 25 simulated matrices with varying levels of noise. For sparsity and downsampling analysis matrices were manually created based on matrices from HiCToolsCompare matrices with the minimum noise level (see Cresswell et al., 2019 for methods description). Data for comparisons across cell lines, replicates, and tissues were taken from (Schmitt et al., 2016), generated at 40 kb resolution (Supplementary Table 1). Time course data was taken from (Rao et al., 2017), HCT-116 human colon cancer cell-line at four time points after auxin-treatment withdrawal (20, 40, 60, 180 min). Contact matrices were generated at 25, 50, and 100 kb using the straw tool from Juicer (Durand et al., 2016). Chromatin state data were taken from chromHMM (Ernst and Kellis, 2010). Histone modifications and transcription factor binding sites were downloaded from the Encyclopedia of DNA Elements (ENCODE) (Davis et al., 2018) (Supplementary Table 2). Scripts to recreate the results presented in the paper are available at https://github.com/cresswellkg/TADCompare_Paper. The TADCompare R package is freely available on GitHub (https://github.com/dozmorovlab/TADCompare) and on Bioconductor (submitted).

## 3. Results

### 3.1. A Modified Spectral Clustering Approach Is Better Suited for Boundary Detection Than Other Approaches

Our previous work on TAD detection using spectral clustering, implemented as a SpectralTAD R package (Cresswell et al., 2019), introduced the concept of the boundary score statistic, adapted here for differential boundary detection. Briefly, the boundary score is calculated for each bin by sliding a window across the diagonal of the contact matrix, calculating the eigenvectors of the Laplacian matrix, finding the distance between consecutive eigenvectors (eigenvector gap) and converting them into Z-scores (boundary score, see Methods). The boundary score is a continuous measure of the likelihood of a given region being a boundary between interacting domains.

In contrast to other metrics for boundary identification that rely on finding inflection points of monotonic functions, such as directionality index (Dixon et al., 2012), insulation score (Crane et al., 2015), RobusTAD score (Dali and Blanchette, 2017) (Supplementary Material), our boundary score spikes at the boundary (Figure 1). This unique behavior enables easy distinction between boundaries and non-boundaries. An additional advantage of the boundary score is that its magnitude is directly interpretable as a “boundary strength.” This is in contrast to other methods which are only interpretable relative to neighboring points. We can use this interpretability for parametric modeling of boundary behavior. Our previous work has shown that the boundary score is robust to noise, sparsity, and changes in sequencing depth of Hi-C data (Cresswell et al., 2019). Thus, the boundary score is well-suited for finding differences in boundaries between interacting domains.

### 3.2. Differential Boundary Scores Translate to Five Types of Boundary Changes

Differential boundary score is a measure of the difference between boundaries between two samples. This score follows a standard normal centered at 0 (see Methods, Supplementary Figure 1). Differential boundaries are detected by finding regions with the absolute differential boundary score is >2 (Supplementary Figure 2), which intuitively corresponds to differences with a *p*-value smaller than 0.05.

**Figure 2:**
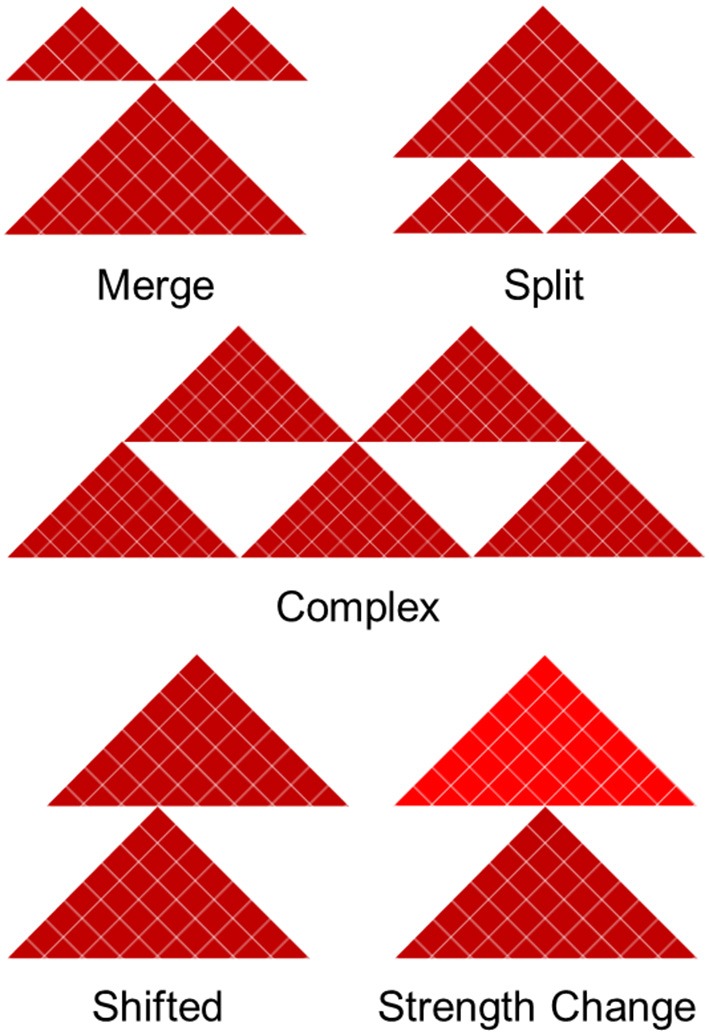
Five types of boundary changes. Complex, split, and merge boundary changes are considered as the major differences, while shifted and strength changes are considered as the minor differences.

We divide boundary changes into five categories (complex, split, merge, shifted, strength change; Figure 2, Supplementary Figure 3). A similar strategy was used in Ke et al. (2017). An interacting domain can be **split** between the datasets, meaning it exists as a continuous domain in one and is split into two or more domains in another. In practice, this situation requires two shared boundaries and a differential domain between them. **Merging** is the opposite of splitting and arises when a boundary surrounded by two non-differential boundaries disappears in one of the contact matrices. Classification of boundary change as merged and split depends on the reference contact matrix being compared to. Finally, domains can be split in a **complex** way, meaning they are neither split or merged but instead taking on an entirely new structure. Merged and split boundaries represent the structural change of the same domain as opposed to complex boundaries, which we consider to be part of a completely different domain. The “complex,” “merge,” and “split” boundaries are considered to be the most disruptive changes in the 3D structure of the genome.

A **shifted** boundary is defined as the non-overlapping boundary that lies within five bins (or another user-defined threshold) of a boundary in the contact matrix in which it is being compared to. A **strength change** occurs when a boundary is present in both contact matrices, but its differential boundary score magnitude is greater than the differential threshold of 2. The other cases are considered to be non-differential boundaries. This framework allows us to systematically compare and classify boundary changes.

### 3.3. Boundaries Are Highly Consistent in Both Technical and Biological Replicates

**Figure 3:**
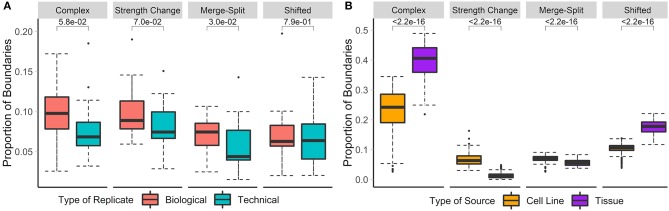
Biological replicates and cell lines have more differential boundaries than technical replicates and tissues, respectively. Differential boundaries were calculated between Hi-C datasets of biological and technical replicates [**A**, HCT-116 cell line, 50 kb resolution, chr 1–22 (Rao et al., 2017)] and between cell lines and tissues [**B**, various cell lines, 40 kb resolution, chr 1–22 (Schmitt et al., 2016)]. Types of boundary changes were recorded, and the proportions of boundary differences for each type were summarized across chromosomes.

Previous studies have shown that the overlap between TAD boundaries in replicate data ranges from around 60 to 70% (Dixon et al., 2012; Rao et al., 2014; Sauerwald et al., 2020). Additionally, technical replicates have been shown to have a slightly higher proportion of shared TAD boundaries (~65%) than biological replicates (~60%) (Sauerwald et al., 2020). We have tested and confirmed these observations by showing that significantly more boundaries were non-differential in technical replicates than in biological replicates (73 vs. 65.7%). Similarly, 9.3/8.1% of boundaries showed significant strength change, while 7.8/6.1% were shifted in the biological/technical replicates, respectively. A similar trend was observed for complex and merge-split boundaries. In summary, only 17.2/12.8% of boundaries were differential in biological/technical replicates, respectively (Figure 3A), confirming the higher stability of the 3D structures in technical replicates.

### 3.4. Boundaries Are More Similar Within Cells Than Tissues

Previous research showed that TADs are largely invariant across cell lines and, to a lesser extent, tissue types (Pope et al., 2014; Rao et al., 2014; Schmitt et al., 2016). However, the types of boundary changes remained undefined. We compared Hi-C matrices of seven different cell-lines and 18 different tissue types (Schmitt et al., 2016) (Supplementary Table 3). In total, the average percentage of differential boundaries was significantly less in cell lines (22.5%) than tissue samples (39.7%, Figure 3B). As expected, these percentages were higher than those for biological (17.2%) and technical replicates (12.8%). These results suggest that the variability of boundaries mirrors the homogeneity of data types (technical replicates, biological replicates, cell lines, and tissues, in that order).

### 3.5. Each Type of Differential Boundaries Is Associated With Different Levels of Epigenomic Enrichment

To understand the biological relevance of the types of boundary changes, we identified changes between the GM12878 and IMR90 cell lines [chr 1–22, 40 kb resolution (Schmitt et al., 2016)] and categorized them according to the type of change. For each change type, we assessed the number of overlapping peaks and calculated the enrichment of four genome annotation marks known to co-locate with TAD boundaries—CTCF, RAD21, insulators, and heterochromatin states.

**Figure 4:**
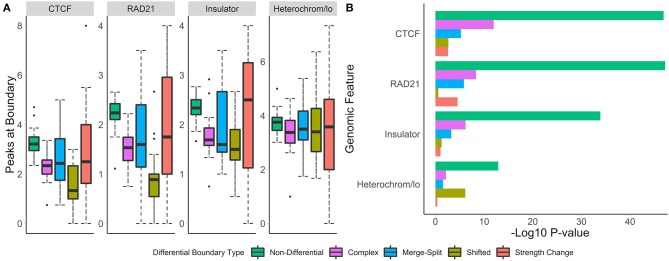
Non-differential boundaries are more enriched for selected genome annotation marks than other types of differential boundaries. Differential boundaries were called between GM12878 and IMR90 cell lines and categorized based on differential boundary type. **(A)** The number of peaks at boundaries and **(B)** permutation *p*-values (−log10) are shown. Data from Schmitt et al. (2016), 40 kb resolution, chr 1–22.

We found that non-differential boundaries had a higher average number of overlapping peaks for all four marks, followed by “strength change” boundaries (Figure 4A). Similarly, enrichment of non-differential boundaries was the most significant (Figure 4B). Notably, the number of peaks for each mark was highly variable on “strength change” boundaries (Figure 4A), suggesting their biological relevance is less certain. Similarly, “shifted” boundaries had the lowest average number of peaks, suggesting that they may be detected due to noise and, consequently, be less biologically significant. In contrast, “complex” and “merge-split” boundaries had a moderate number of overlapping peaks and were moderately enriched in them (Figure 4). These results highlight the varied biological relevance of different types of boundary changes and suggests “complex” and “merge-split” changes are biologically important alterations of the 3D structure.

### 3.6. Each Type of Differential Boundaries Is Associated With Distinct Biological Functionality

**Figure 5:**
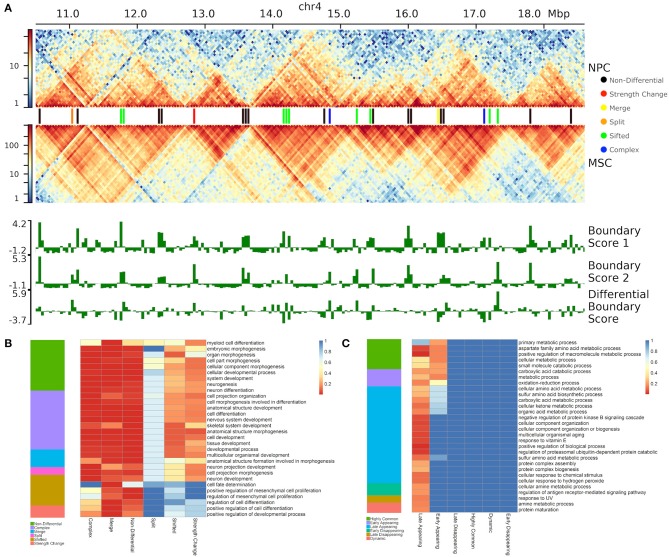
Differential boundaries and their gene enrichment analysis. **(A)** An example of differential boundaries called between neural progenitor cell (NPC) and mesenchymal stem cells (MSC) (Schmitt et al., 2016) (chr4:10500000–18600000 region, 40 kb resolution); outlined TADs were called using SpectralTAD (Cresswell et al., 2019). **(B,C)** The top 30 gene ontologies most enriched **(B)** in NPC vs. MSC boundary comparison, and **(C)** across the time-course of boundary changes in auxin-treated cells from the HCT-116 cell-line (Rao et al., 2017) (chr 1–22, 40 kb resolution). For each type of boundary change, enrichment *p*-values (rGREAT, see Methods) are shown as heatmaps.

To test the biological significance of different types of boundary changes, we compared neural progenitor cells (NPC) against mesenchymal stem cells (MSC) (Schmitt et al., 2016) (Figure 5A, Supplementary Figure 3C). Altogether, we found that the vast majority of boundaries are either complex (38.6%) or non-differential (32.6%). Shifted (17.5%), merge-split (7.7%) and strength change (3.5%) were less common (Figure 5B). Under the hypothesis that differential boundaries may be enriched in genes driving relevant biological processes (Chen et al., 2018), we investigated enrichment of genes in proximity of each type of differential TAD boundary in biological processes and other gene ontology- and pathway types using GREAT (McLean et al., 2010) (see Methods). As NPCs are more advanced on differentiation path than MSCs, we expected that boundaries changed between them would be associated with genes responsible for neural development-related processes. Indeed, genes around “merge” and “complex” boundary changes, as well as the “non-differential” boundaries were enriched in a variety of developmental processes (e.g., “cellular developmental process,” etc.), including neural-specific (“nervous system development,” Figure 5B). Notably, “split” boundary changes were not enriched in these processes, indicating the importance of the directionality of boundary changes. Genes around “merge” and “non-differential,” but not “complex,” boundaries were enriched in differentiation-related processes (e.g., “positive regulation of cell differentiation”), while “forebrain radial glial cell differentiation” and “neural tube development” processes were exclusively enriched in genes around “merged” boundaries (Figure 5B). In this case, “merge” indicates boundaries enriched in the NPC cell-line, causing a separation of interacting domains in MSC and “split” indicates a split in NPC caused by a boundary enriched in MSC. As expected, genes around “noisy” boundary changes (“shifted” and “strength change”) lacked enrichment in any biological processes (Figure 5B, Supplementary Table 4). These results emphasize the importance of classifying boundary changes into distinct patterns that tend to be associated with distinct biological functionality.

To further test whether different types of boundary changes reflect biology of an experimental system, we used post-auxin treatment time course experiment from Rao et al. (2017) study (HCT-116 cell line, 40 kb resolution, 20, 40, 60, and 180 min following auxin withdrawal, 4 replicates at each time point) (Rao et al., 2017). Auxin treatment eliminates CTCF binding genome-wide; consequently, the majority of boundaries should be absent and gradually re-appear following auxin withdrawal. To identify biological processes associated with re-appearing of boundaries, we compared first and last time points (20 and 180 min) following auxin withdrawal. As boundaries were reported to be enriched in housekeeping genes (Jin et al., 2013), we expected genes around appearing boundaries to be enriched in general cellular processes. Indeed, the vast majority of boundaries were complex (41.4%) and non-differential (34.7%) (Supplementary Figure 4). We found that only genes around “non-differential” and “complex” TAD boundary changes showed some level of enrichment (Supplementary Figure 4, Supplementary Table 5). As expected, “metabolic processes” and various developmental and housekeeping processes were specifically enriched in genes around complex boundary changes, while cyclic AMP synthesis and metabolic processes were enriched in genes around “non-differential” boundaries. From these results, we show that TADCompare can correctly classify less-essential boundary changes (“shifted,” “strength change”) and detect distinct boundary changes associated with shared and unique biological processes.

### 3.7. Time Course Analysis Framework

Time course analysis of boundaries refers to the analysis of boundary dynamics over time. The quantitative nature of boundary score allows us to monitor its changes at boundaries across any number of time points. We recommend taking a union of boundaries detected at each time point and monitor boundary score changes for each boundary. Monitoring boundary scores across time points provides an opportunity to quantify patterns of boundary changes.

**Figure 6:**
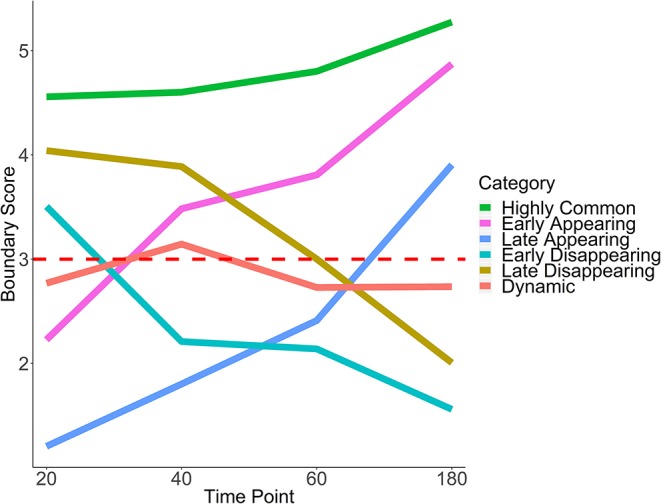
Six patterns of boundary score change across time. Average trajectories for each pattern of boundary score change are shown. The red horizontal line indicates the cutoff for boundary detection. HCT-116 cell line, 40 kb resolution, chr 1–22.

Using the boundary score cutoff of 3 for boundary definition, we define six patterns of temporal boundary changes (adapted from Zhou et al., 2019, Table 1, Figure 6). *Highly common* boundaries refer to boundaries present across all time points or in three out of four time points. *Early appearing* boundaries switch from non-boundary to boundary at second time points and stay as boundaries for the rest of the time points. Conversely, *early disappearing* boundaries switch from boundary to non-boundary at the second time point and stay as non-boundaries. *Late appearing* boundaries switch from non-boundaries to boundaries at the last or the second to last time point. Conversely, *late disappearing* switch from boundaries to non-boundaries at the last of the second to last time point. Finally, *dynamic* boundaries are those which have inconsistent boundary status and do not follow any of the aforementioned patterns (Figure 6). These six patterns of temporal changes can be easily adapted for a larger number of time points.

**Table 1:** Six patterns of temporal boundary changes.

**Temporal boundary type**	**Time point 1**	**Time point 2**	**Time point 3**	**Time point 4**	**Total (% occurrence)**
Highly common	1	1	1	1	326 (17.35%)
	1	0	1	1	
Early appearing	0	1	1	1	184 (9.79%)
Early disappearing	1	0	0	0	133 (7.08%)
Late appearing	0	0	1	1	1,047 (55.72%)
	0	0	0	1	
Late disappearing	1	1	0	0	79 (4.20%)
	1	1	1	0	
Dynamic	1	0	1	0	110 (5.86%)
	1	0	0	1	

*Each column corresponds to a point in time*.

*“1” refers to the presence of a boundary, and “0” refers to the absence of a boundary. The “Total” column shows the percentage of occurrences in the CTCF degradation-recovery time course, HCT-116 cell line, chr 1–22 (Rao et al., 2017)*.

### 3.8. Temporal Boundary Types Are Associated With Different Levels of Epigenomic Enrichment

To evaluate the biological relevance of temporal patterns of boundaries, we used post-auxin treatment time course experiment introduced above. Briefly, HCT-116 cells were treated with auxin to eliminate boundaries, and Hi-C measures were obtained at 20, 40, 60, and 180 min following auxin withdrawal and subsequent boundary reappearance (Rao et al., 2017). Accordingly, we expected to detect some number of highly common boundaries (already existing at 20 min) and boundaries appearing at different stages of post-auxin withdrawal (early/late appearing). Conversely, dynamic and early/late disappearing boundaries should be rare and may potentially constitute noise in TAD boundary detection.

Boundary scores were calculated for auxin-treated cells 20, 40, 60, and 180 min after withdrawal. Taking the union of boundaries (boundaries detected at one or more time points), we calculated temporal patterns for each boundary. We found that the vast majority of boundaries were late appearing (55.7%) (Table 2, Figure 5C). Early appearing (9.8%) and highly common (17.3%) made up most of the other boundaries present at the end of the time course. Approximately 20% of boundaries were highly common, i.e., resistant to auxin treatment, a number similar to previous works (Nora et al., 2017). Meanwhile, 5.9% of boundaries were dynamic, 7.1% were early disappearing, and 4.2% were late disappearing, highlighting potential errors in boundary detection. In summary, some boundaries can be detected at 20 min post-auxin treatment and remain present through all time points; however, the timing of boundary reappearance varies.

**Table 2:** Consensus (aka median) boundary score is supported by high boundary scores from multiple replicates.

**Boundary score 1**	**Boundary score 2**	**Boundary score 3**	**Consensus boundary score**	**Union boundary?**	**Consensus boundary?**
1	2	1	1	No	No
3	2	1	2	Yes	No
5	5	4	5	Yes	Yes
3	3	3	3	Yes	Yes
6	0	0	0	Yes	No

*Examples of boundary scores across five regions in three replicates, and the corresponding consensus boundary score. Both union and consensus boundaries are calculated using a cutoff of 3*.

**Figure 7:**
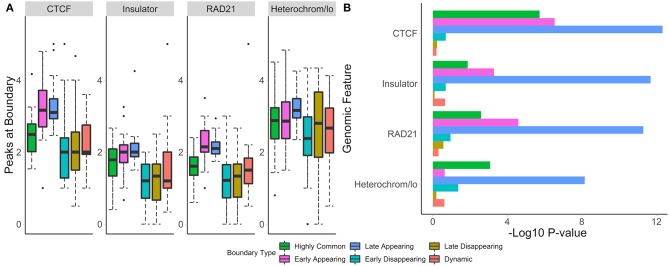
Common and appearing boundaries show stronger enrichment in known epigenomic marks. The number of peaks at boundaries **(A)**, and permutation *p*-values **(B)** within 50 kb of boundaries in each temporal classification are shown. Hi-C data from Rao et al. (2017), 50 kb resolution, HCT-116 cell-line, chr 1–22.

To test whether boundaries associated with different temporal patterns have different functional roles, we investigated their overlap with and enrichment in the common marks of TAD boundaries (CTCF, RAD21, insulators, heterochromatin, Figure 7A). For highly common, early- and late-appearing boundaries, we observed more overlaps with CTCF and RAD21 sites, insulator, and heterochromatin states (Supplementary Table 6). Similarly, these types of boundaries were highly enriched in the aforementioned genomic annotations (Figure 7B). Conversely, dynamic, early, and late disappearing boundaries showed less overlap with CTCF, RAD21, insulator, and heterochromatin marks, and were less enriched in them. These observations suggest that disappearing and dynamic boundaries are likely detected due to noise in the data, while boundaries appearing after auxin treatment expectedly represent the biologically relevant signal.

### 3.9. Temporal Boundary Types Are Associated With Distinct Biological Functionality

Using gene enrichment analysis, we further investigated whether boundaries associated with different temporal patterns may be enriched in genes driving relevant biological processes (Chen et al., 2018) (Supplementary Table 7). We found that, with a few exceptions, all significant GO Biological pathways were enriched in late or early appearing boundaries (Figure 5C, Supplementary Table 7), which make up the majority of boundaries (Table 2, Figure 5C). Both early and late appearing boundaries were enriched in metabolism-related processes, such as “cellular metabolic process,” “oxidation-reduction process.” Late appearing boundaries, on the other hand, were enriched in “cellular component organization,” “protein complex biogenesis” and the like processes (Figure 5C). These results are expected as cells may be activating metabolic and biogenesis pathways to recover after destruction of boundaries by auxin. These results confirm that TADCompare can accurately classify biologically relevant temporal boundary changes and discern them from noisy changes.

### 3.10. Consensus Boundary Score for Defining Robust Boundaries Across Multiple Hi-C Datasets

The sizeable proportion of noisy “shifted” and “strength change” boundary changes across Hi-C datasets (Figure 3) highlights the need to identify boundaries that are robustly detected. The consensus boundary score, defined as the median of boundary scores across replicates, addresses this challenge. Intuitively, higher consensus boundary scores correspond to boundaries supported by evidence from multiple replicates (Table 2). This is in contrast to a union of boundaries, where boundaries detected in at least one Hi-C dataset are pooled together. Consensus boundary scores allow us to filter out boundaries with insufficient support from multiple replicates, thus “denoise” the detected boundaries. Given the fact that boundary scores are log-normally distributed (Supplementary Figure 1, Supplementary Methods), the consensus boundary scores will also be asymptotically normal. The consensus boundary score can be used as a proxy for the normal boundary score for the analysis of replicated Hi-C datasets. Consequently, the consensus boundary scores may be compared to define boundary changes between groups of replicated Hi-C datasets.

### 3.11. Consensus Boundaries Are Supported by Strong Biological Evidence

**Figure 8:**
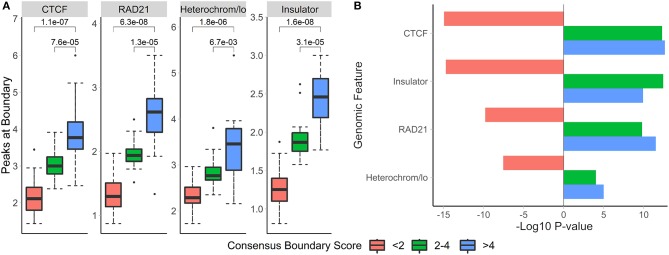
Boundaries defined at higher consensus boundary score thresholds show stronger overlap with and enrichment in known epigenomic marks. Boundaries were classified based on the range of their consensus boundary score. Enrichment of genomic factors known to occur near TAD boundaries was calculated. **(A)** The number of peaks within 40 kb of boundaries with the corresponding consensus score range and **(B)** the −log10-transformed permutation *p*-values for each score range are shown. Negative *p*-values indicate depletion. Data from seven cell lines, chr 1–22, 40 kb resolution (Schmitt et al., 2016).

To investigate the biological relevance of boundaries defined using consensus boundary score, we defined consensus boundaries across seven cell-lines (17 matrices total) (Schmitt et al., 2016). These boundaries represent cell type-invariant boundaries supported by evidence from multiple datasets. Bins of the genome were separated into three categories based on the level of their consensus boundary score (<2, 2–4 and >4). In total, there were 65,336 bins (40 kb resolution). Expectedly, the majority (62,791 bins, 96.1% of all bins) were in the <2 category, 2,032 (3.1%) bins were in the 2-4 category, and 513 (0.8%) bins were in the >4 category. We assessed the number of overlapping peaks and the enrichment of CTCF, RAD21, insulators, and heterochromatin states in different categories of bins. Expectedly, we observed increasing average number of peaks overlapping bins selected at more stringent consensus boundary score thresholds (Figure 8, Supplementary Table 8). Similarly, bins with higher consensus boundary scores have stronger enrichment in genome annotations, while bins with score <2 were significantly depleted. These results suggest that bins with higher consensus boundary scores (i.e., supported by evidence from multiple Hi-C datasets) are more biologically relevant. Therefore, to define consensus boundaries, we use a consensus boundary score cutoff of 3.

### 3.12. The Union of Boundaries Is Supported by Weaker Biological Evidence Than Consensus Boundaries

The union of boundaries called in individual Hi-C datasets represents an alternative method of defining boundaries across multiple datasets (Table 1). The union method may be useful for analysis of time course data, where boundaries are expected to change across individual datasets. We hypothesized that the union method would select for the less biologically relevant set of boundaries because many may be detected due to noise in Hi-C data.

**Figure 9:**
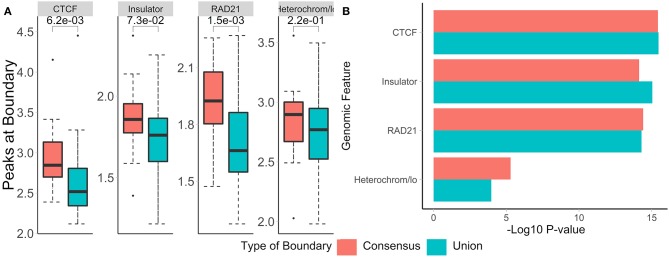
Consensus boundaries show stronger overlap with and enrichment in known epigenomic marks than the union of boundaries. **(A)** Number of peaks at boundaries and **(B)** permutation *p*-values (−log10) are shown. Data from Schmitt et al. (2016), four cell lines, 40 kb resolution, chr 1–22.

To evaluate the biological relevance of boundaries called using both methods, we call consensus and union boundaries on a set of replicates (four cell lines, 40 kb resolution, three replicates each, data from Schmitt et al., 2016). Consensus scores were calculated separately for each cell line among the three replicates. Expectedly, the consensus method filtered out 38% of boundaries (4,906 vs. 3,059, Supplementary Figure 5), suggesting that many boundaries are detected in single datasets. We found that boundaries called using consensus boundary score overlapped significantly more with CTCF sites (P = 0.0006) and RAD21 (P = 0.0002) than those called using the union method (Figure 9A). While the enrichment results were similar for consensus- and union-defined boundaries, consensus boundaries were more significantly enriched in “heterochromatin” (Figure 9B). Together with previous observations (Figure 6), these results strengthen our conclusion that consensus boundary scores are more effective in removing “noisy” boundaries that otherwise would be captured using the union method.

### 3.13. Runtime Performance of TADCompare

When run on data from (Rao et al., 2014), without parallelization, both consensus boundary calling and differential boundary detection were exceptionally fast. In total, for the entire genome, differential boundary detection took ~6 s on 100 kb data, ~9 s on 50 kb data, ~17 s on 25 kb data, and ~312 s on 10 kb data. In the case of consensus boundary calling, TADCompare took ~17 s to run on 50 kb data for 4 matrices, ~32 s for 8 matrices, and ~45 s for 12 matrices. On 10 kb data, it took ~611 s to run for 4 matrices, ~1,152 s for 8 matrices, and ~1,680 s for 12 matrices. For a full summary of runtimes across all resolutions (see Supplementary Figure 6).

## 4. Discussion

The initial development of Hi-C technologies focused on investigating individual genomes. While several key properties have been discovered (chromosome territories, A/B compartments, TADs, chromatin loops, collectively referred to as “interacting domains”), the next steps include investigating changes in the 3D structure across multiple conditions. We (Stansfield et al., 2018, 2019) and others (Lun and Smyth, 2015; Djekidel et al., 2018) started to develop methods for comparative analysis of the 3D structures. However, to our knowledge, no methods are available for differential analysis of boundaries demarcating interacting domains. In this work, we introduce a method for differential boundary analysis, including a time course, that supports replicated Hi-C data. The method is based on a novel boundary score metric that provides a continuous measure of boundary likelihood (Cresswell et al., 2019). We introduce unique terminology for classifying differential and temporal boundary changes. We show that our approach is robust and effective at identifying distinct biology associated with different types of boundary changes. Our method is implemented in the TADCompare R package available on Bioconductor, filling a vital gap in intuitive R-based software for boundary detection and comparison.

The boundary score concept developed in our work addresses three main problems: differential boundary detection, time course analysis of boundary changes, and consensus boundary calling. Yet, it has a broader scope of applications. Future work will expand the utility of boundary score by developing a similarity/reproducibility score to measure the agreement between (multiple) Hi-C matrices, in the same vein as HiCRep (Yang et al., 2017), Selfish (Ardakany et al., 2019), GenomeDISCO (Ursu et al., 2018), HiC-Spector (Yan et al., 2017), QuASAR-Rep (Sauria et al., 2015). Furthermore, for differential boundary detection, our method is still limited to the comparison of two profiles of (consensus) boundary scores. This approach will eventually be expanded to include comparisons of many contact matrices, similar to the concept of comparing groups of multiple replicates in RNA-seq data. Finally, there is still room for expansion of time course boundary analysis. The continuous nature of boundary score allows for adopting time course analysis methods developed for gene expression studies (Bar-Joseph et al., 2012). More flexible classification of temporal trends may be considered, such as 24 temporal patterns proposed by Zhou et al. (2019), or fuzzy clustering techniques that do not require a pattern to belong to a specific cluster (Abu-Jamous and Kelly, 2018). In summary, our work enables further development of various aspects of 3D genome analysis.

One difficulty in our work is how to accurately quantify the biological relevance of boundaries (differential, time-varying, and consensus) that we detect. There is no natural gold standard for boundaries, but there are known genomic features that form the building blocks of TADs (CTCF, RAD21). In practice, we can use colocalization and/or signal enrichment of these marks near boundaries as a proxy for “true boundaries.” To test whether enrichment is different than random (non-boundaries), we use a permutation test and present these *p*-values. In the current work, we used colocalization enrichment analysis, and plan to address changes in signal enrichment associated with changes in boundaries in future work.

The goal of the TADCompare package is to provide a practical implementation of our statistical framework for differential boundary detection. It outputs genomic coordinates of differential boundaries, type of the differences, and the associated boundary score measures. The downstream analysis options may be gene enrichment analysis in the proximity of (different types of) differential boundaries using rGREAT, epigenomic enrichment analysis [GenomeRunner (Dozmorov et al., 2012, 2016), LOLA (Sheffield and Bock, 2016)], and visual exploration of differential boundaries. Although TADCompare provides simultaneous visualization of two Hi-C matrices and the associated boundary differences and boundary scores, external tools for visualizing multiple datasets may be explored (reviewed in Yardimci et al., 2019). Tools like the HiCBricks R package (Pal et al., 2019) and the HiCexplorer Python software (Ramirez et al., 2018) start enabling the users to visualize two Hi-C matrices and the associated annotations. We continue exploring visualization options to improve exploration and interpretation of boundary differences.

Our results in this manuscript demonstrate the ability of TADCompare to provide accurate, biologically relevant results. The methods implemented span differential, time-course, and consensus analysis. To date, TADCompare is the only actively maintained and publicly available tool to provide any of this functionality. We intend for TADCompare to be a one-stop tool for comparison of HiC datasets, providing simple, easy-to-interpret results in a timely manner. As a one-of-a-kind tool, TADCompare will increase the ability of researchers to extract important biological insights from the structure of TAD boundaries.

## Data Availability Statement

Publicly available datasets were analyzed in this study. This data can be found here: All URLs are listed in the Supplementary Tables 1, 2. Main datasets include: ftp://ftp.ncbi.nlm.nih.gov/geo/series/GSE87nnn/GSE87112/suppl/GSE87112_file.tar.gz, https://bitbucket.org/mforcato/hictoolscompare/get/406ee2349566.zip, https://www.ncbi.nlm.nih.gov/geo/download/?acc=GSE104333, https://www.ncbi.nlm.nih.gov/geo/download/?acc=GSE63525.

## Author Contributions

MD and KC conceived the project. KC implemented the TADCompare and wrote the analysis scripts. MD and KC wrote the manuscript.

### Conflict of Interest

The authors declare that the research was conducted in the absence of any commercial or financial relationships that could be construed as a potential conflict of interest.

## Supplementary Material

Supplementary Figure 1 Log-normal distribution of eigenvector gaps converted to boundary Z-scores. Eigenvector gaps were calculated for contact matrices across three resolutions [10, 25, and 50 kb, Hi-C data from Rao et al. (2014), GM12878 cell line, chr 1–22]. Density plots are shown for the **(A)** Natural log of the eigenvector gaps and **(B)** Boundary scores derived from the same data, separated by resolution. Regions of non-TADs are highlighted by a yellow bar, moderate strength boundaries (2 < boundary score cutoff <3) are highlighted by a red bar, and strong boundaries (cutoff > 3) are shown using a green bar. We see a slightly right-skewed distribution due to the filtering of gaps for plotting purposes.

Supplementary Figure 2 Window size of 15 units of Hi-C data resolution and boundary score cutoff of 2 yields consistent boundary detection. Differential boundaries were compared between two simulated data sets with window size sizes ranging from 10 to 25, and boundary score cutoff ranging from 1.5 to 4. Youden index (balanced sensitivity and specificity metric) was calculated for each combination and plotted to show agreement with ground-truth annotations. Results are shown for noise-injected matrices **(A)** and sparsity-injected matrices **(B)**.

Supplementary Figure 3 Visualization of different types of boundary score patterns. **(A)** Patterns of raw boundary scores are shown for five different types of differential boundaries (Merge, split, complex, shifted, and strength change). The red horizontal line corresponds to the user-adjustable cutoff for a boundary. Human neural progenitor cells (NPCs), chr22, most representative examples are shown. **(B)** TADCompare::DiffPlot differential boundary visualization between NPCs and mesenchymal stem cells (MSC), chr4:10500000–18600000. 40 kb resolution data from Schmitt et al. (2016).

Supplementary Figure 4 Heatmap of gene ontology enrichment at the first and last time point in auxin-treated data. Differential boundary identification was performed on auxin-treated data at the time of application (first time point) and complete withdrawal (last time point) [HCT-116 cell line, chr 1–22, 40 kb resolution (Schmitt et al., 2016)]. A barplot of the proportion of each differential boundary type and FDR-adjusted hypergeometric *p*-values obtained from gene ontology enrichment analysis using rGREAT (see Methods) are shown. The top 30 pathways, in terms of average enrichment, are shown and clustered using the Ward method.

Supplementary Figure 5 Venn diagram of union and consensus boundary counts. Consensus and union boundaries were called across four different cell lines (hesc, mesynchymal, npc, trophectoderm), and the number of union and consensus boundaries was recorded. The Venn diagram shows the complete overlap of consensus boundaries within union boundaries (40 kb resolution, data from Schmitt et al., 2016).

Supplementary Figure 6 Runtime of TADCompare. Plot containing the runtime of two-way comparison **(A)** and consensus boundaries called on 4, 8, 12, and 16 replicates **(B)**. Each point represents the runtime for a specific chromosome. X-axis—chromosome, Y-axis—runtime in seconds. Hi-C data from Rao et al. (2014), chr 1–22, 10, 25, 50, and 100 kb resolution.

Supplementary Table 1 Contact matrix data sources. The source of all contact matrices, experimental, and simulated, used in this paper are provided. Experimental data are separated based on the study and cell line.

Supplementary Table 2 Genomic annotation data sources. The sources, with download links, for all genomic annotations used in this paper are included.

Supplementary Table 3 Summary of differential boundary types across tissues and cell lines. The percentage of each type of differential boundary for all tissue-tissue and cell line-cell line comparisons is reported. Results are aggregated over all chromosomes. Hi-C data from Schmitt et al. (2016), 40 kb resolution, chr 1–22.

Supplementary Table 4 Gene ontology enrichment for differential boundary types. Differential boundaries were identified between the neural progenitor cells (NPC) and mesenchymal stem cells (MSC) (Schmitt et al., 2016). Pathway analysis was performed using rGREAT (Methods), and results are separated by ontology. Boundaries with an FDR adjusted *p*-value of <0.3 are shown. 40 kb resolution, chr 1–22.

Supplementary Table 5 Gene ontology enrichment between the first and last time points in auxin-treated data. Differential boundaries were identified between the first and last time points of auxin-treated data (Rao et al., 2017). Pathway analysis was performed using rGREAT (Methods), and results are separated by ontology. Boundaries with an FDR adjusted *p*-value of <0.3 are shown. 50 kb resolution, chr 1–22.

Supplementary Table 6 Enrichment across different temporal boundary types. Temporal boundary types were identified across four time points in auxin-treated data (Rao et al., 2017). Results are shown for four types of temporal boundaries (Early Appearing, Late Appearing, Highly Common, Dynamic). Permutation *p*-values, along with enrichment or depletion designations, are reported. HCT-116 cell line, 40 kb resolution, chr 1–22.

Supplementary Table 7 Gene ontology enrichment for different temporal boundary types. Temporal boundary types were identified across four time points in auxin-treated data (Rao et al., 2017). For each temporal boundary type, pathway analysis was performed using rGREAT (Methods), and results are separated by ontology. Boundaries with an FDR adjusted *p*-value of <0.3 are shown. HCT-116 cell line, 50 kb resolution, chr 1–22.

Supplementary Table 8 Enrichment across different consensus scores. Consensus scores were called across 17 contact matrices representing seven different cell lines. Results were dichotomized into three groups (<2, 2–4, >4) based on consensus boundary scores. Permutation *p*-values, along with enrichment or depletion designations, are reported. Hi-C data from Schmitt et al. (2016), 40 kb resolution, chr 1–22.
