# Supplementary material for: TADCompare: An R Package for Differential and Temporal Analysis of Topologically Associated Domains
Source: Front Genet. 2020 Mar 10;11:158. doi: 10.3389/fgene.2020.00158 (PMC7076128; doi:10.3389/fgene.2020.00158)
Supplement: Supplementary file 1 [file Presentation_1.pdf]

Supplementary Material

Supplementary Methods

Previous methods

Traditionally, TADs have been compared by overlap-based metrics, such as Jaccard index and Venn diagrams (Dixon et al. 2012; Rao et al. 2014). These methods provide important information but suffer from inability to capture the dynamics of individual TAD boundaries, instead providing a global measure of TAD similarity. Additionally, they are heavily reliant on the method used to call TADs. This reliance exposes them to the large variation in TAD caller quality (Forcato et al. 2017; Dali and Blanchette 2017; Zufferey et al. 2018). To date, no method exists that can identify and classify differential TAD based on their comparative structure.

DiffTAD (Zaborowski and Wilczynski 2016) was the first publicly available method for direct comparison of TADs, at a TAD-by-TAD level. Their method works by taking two contact maps with pre-defined TADs, creating a differential contact map by subtracting the matrices from each other, subsetting the contact matrix based on TAD locations and either doing a parametric or non-parametric test on the differential contact map. The idea is that large values in the differential contact map correspond to differential regions. DiffTAD require users to specifically run the Armatus (Filippova et al. 2014) TAD caller before analysis, or at the very least format their data to match the output of Armatus. Armatus is known to be sensitive to different parameters, resolution and sequencing depth (Dali and Blanchette 2017), thus hindering unambigious TAD detection. Consequently, DiffTAD performance and user experience may be unsatisfactory.

Sauerwald et al. developed a method for quantifying the similarity of two sets of TADs using variation of information (VI) metric (Sauerwald and Kingsford 2018). VI is a general information theoretic metric for computing distance between two clusterings; in terms of Hi-C data, a cluster is a set of Hi-C bins placed in the same TAD. This approach is significant in that it provided the first continuous measure of TAD similarity at the boundary level. In their method, VI is calculated at TAD boundaries and a permutation test is used to determine cutoffs for differential regions. This method, referred to as TADsim was followed by an updated version called localTADsim (Sauerwald, Singhal, and Kingsford 2018). The new method is designed to be fast and introduces the concept of "hanging TADs". Hanging TADs refer to TAD boundaries which start within other TADs. `localTADSim` ignores these TADs despite previous research highlighting the biological importance of such TADs (Weinreb and Raphael 2016; Fraser et al. 2015; Gibcus and Dekker 2013; Dong et al. 2018; Cresswell, Stansfield, and Dozmorov 2019). Like DiffTAD, `localTADsim` requires users to use Armatus or to manually format their inputs to look like Armatus output. Additionally, this method has been shown to be exceptionally slow with speed depending on the number of TADs analyzed. Examples show run times of around an hour for certain chromsomes at 100kb resolution (Sauerwald and Kingsford 2018), creating problems with reproducibility and user

experience like for DiffTAD.

The latest method to be introduced, HiCDB (Chen et al. 2018), uses a metric called relative local insulation (RI). This metric is similar to insulation score (Crane et al. 2015) but includes terms for correcting for background noise. The method works by detecting TADs between two contact matrices and then calculating the difference in relative local insulation (RI). The differential TAD boundaries are detected as any boundary where the difference in RI values is above the 90% quantile of RI differences. However, this approach is flawed in that it artificially forces 10% of TAD boundary pairs to be detected differential irrespectively of the data properties.

Another criticial issue with these tools are a lack of upkeep. As the resolution (and the corresponding size) of Hi-C data continue to increase, users are often interested in comparing data in individual chromosomes. HiCDB forces users to provide information for all chromosomes simultaneously, which, combined with slow runtime, makes it unsuitable for the analysis of modern Hi-C datasets. DiffTAD has been in a short pre-print form since 2016 and has not been updated since January 2017. In general, the previous methods are slow, require complex data inputs and output results that are difficult to interpret. Thus, a fast, flexible, and user-friendly R package for the detection of differential TADs is needed.

The uniqueness of `TADCompare` in terms of differential detection comes from the complete integration of TAD calling with the differential detection itself. Of the methods listed, only HiCDB is integrated with the TAD caller itself, using its relative insulation score to find differences. By removing the requirement of outside TAD calling, we remove an extra source of noise caused by inconsistent TAD callers and provide a completely data-driven approach.

Another advantage of `TADcompare` is its ability to quantify TAD boundary strength, enabling statistical comparison of them. This is in contrast to methods like `localTADsim`, which does not use data from the contact matrix at all, thus lacking the ability to use statistical methods or quantify the degree of similarity at a given TAD boundary. This limitation requires complete reliance on the chosen TAD caller to account for statistical properties of contact matrices. `diffTAD` differs from our method in that it directly compares contact frequencies within regions bounded by TADs instead of directly analyzing the structure of TADs or their boundaries. In their own words, their approach analyses "differential contact frequency in topological domains" while TADCompare looks directly at differences in boundaries. Compared to other methods,`TADCompare` provides a unique middle-ground, being a data-driven method that has the ability to recognize TAD boundaries without the need for outside TAD callers.

Log-normality of eigenvector gaps allows for calculation of boundary score

Our method relies on the assumption that the distance between eigenvectors is lognormally distributed. We tested this assumption by collecting all distances across all chromosomes and 10kb, 25kb and 50kb. We then fit six potential distributions (gamma, Weibull, lognormal, normal, logistic, Cauchy) with maximum likelihood estimation using `fitdistr` function from

the `MASS` R package (Version 7.3-51.4). This process was performed on (Rao et al. 2014) data. The fit of each distribution was compared using the log-likelihood. Out of the 131 real datasets, 67 were fit best by a lognormal distribution and 64 were fit best by a Weibull. Empirically, we find that in general, the log-likelihoods of these values are similar. Due to the interchangeability of the methods, we choose to model these values as lognormal as this gives us the ability to calculate boundary scores and make statistical inferences on boundary strength.

To allow for direct comparison of eigenvector gaps between contact matrices it is important that their distribution is relatively consistent. If we have large variation in mean or variance between contact matrices' eigenvector gaps then the magnitude of boundary scores can be inflated or deflated making differential detection inaccurate. We assess this by plotting the distributions separated by resolution (Supplementary Figure 1). It is clear that within resolutions, there is little variation in distributions. However, as resolution increases we have many more smaller eigenvector gaps. Between resolutions, the distribution is consistent but as the resolution becomes finer the proportion of non-TADs increase and increase the number of boundary scores around zero. Based on these results, we see that boundary scores behave as expected and can be used as intended

Selecting parameters for differential TAD detection

The definition of boundary score, and the subsequent differential boundary score, requires tuning of two parameters, the size of the sliding window used to calculate eigenvector gaps (see Methods), and the threshold for differential boundary score. The window size has an effect on the actual boundary score value as it controls the distance of contacts considered for the analysis and calculation of the Laplacian matrix. The differential boundary score threshold determines the value of differential boundary score in which we consider a region truly "differential".

To test for the optimal boundary score, we took simulated matrices with known differential TAD locations and added noise and sparsity. `TADCompare` was ran with a range of window sizes and z-thresholds and calculated the ability of algorithm to accurately find true differences. We found that as levels of sparsity and noise increase, a window size of 15 and a threshold value of 2 provided consistent robust results (Supplementary Figure 2).

Selecting the cutoff for consensus boundary score

To dichotomize TADs into TAD boundaries and non-TAD boundaries, one must select a cutoff for the consensus boundary score. There are two approaches to selecting a cutoff: statistical and biological. Since consensus boundary score is effectively a z-score, we can select cutoffs corresponding to popular p-values or critical values. For instance, a cutoff of 1.96 (P = 0.05) or a cutoff of 2.58 (P = 0.01). Another alternative is to use a biologically significant cutoff. Biological significance can be determined by observing the level of enrichment of genomic features, such as CTCF or RAD21 at the TAD boundary, depending on boundary score. In this work, we observe that a cutoff between 2 and 4 achieves a sort of middle ground for significance of genomic features at TAD boundaries (Figure 5). Accordingly, we choose

3 as our TAD boundary cutoff because this ensures we have a reasonably large supply of TADs without sacrificing quality of boundaries. In practice, whether one uses a statistical or biological approach, there is a trade-off where higher score cutoffs reduce noise and increase boundary quality but identify less TAD boundaries. As a result of this trade-off, we set our default in the package itself to 3 and allow users to determine their own cutoff if they desire.

Figures

Supplementary Figure 1. Log-normal distribution of eigenvector gaps converted to boundary Z-scores. Eigenvector gaps were calculated for contact matrices across three resolutions (10kb, 25kb and 50kb, Hi-C data from (Rao et al. 2014), GM12878 cell line, chr 1-22). Density plots are shown for the (A) Natural log of the eigenvector gaps and (B) Boundary scores derived from the same data, separated by resolution. Regions of non-TADs are higlighted by a yellow bar, moderate strength TADs ($2 <$ boundary score cutoff < 3) are highlighted by a red bar and strong TADs (cutoff > 3) are shown using a green bar. We see a slightly right-skewed distribution due to the filtering of gaps for plotting purposes.

Supplementary Figure 2. Window size of 15 units of Hi-C data resolution and TAD boundary score cutoff of 2 yield consistent TAD boundary detection. Differential TAD boundaries were compared between two simulated data sets with window size sizes ranging from 10 to 25 and boundary score cutoff ranging from 1.5 to 4. Youden index (balanced sensitivity and specificity metric) was calculated for each combination and plotted to show agreement with ground-truth annotations. Results are shown for noise-injected matrices (A) and sparsity-injected matrices (B).

Supplementary Figure 3. Visualization of different types of boundary score patterns. Patterns of raw boundary scores are shown for 5 different types of differential boundaries (Merge, split, complex, shifted and strength change). The red horizontal line corresponds to the minimum cutoff for a TAD boundary (40kb resolution, human neural progenitor cell line, data from (Schmitt et al. 2016)). Data from chromosome 22 with the most representative examples chosen.

Supplementary Figure 4. Heatmap of gene ontology enrichment at the first and last time point in auxin-treated data. Differential boundary identification was performed on auxin-treated data at the time of application (first time point) and complete withdrawal (last time point) (HCT-116 cell line, chr1-22, 40kb resolution, (Schmitt et al. 2016)). A barplot of the proportion of each boundary type (A) and FDR-adjusted hypergeomtric p-values (B) obtained from gene ontology enrichment analysis using `rGREAT` (See methods) are shown. The top 30 pathways, in terms of average enrichment, are shown and clustered using Ward clustering.

Supplementary Figure 5. Venn diagram of union and consensus TAD counts. Consensus and union TADs were called across four different cell lines (hesc, mesynchymal, npc, trophectoderm) and the number of union and consensus TADs were recorded. The venn diagram shows the complete overlap of consensus TADs within union TADs. (40kb resolution, data from (Schmitt et al. 2016))

Supplementary Figure 6. Runtime of TADCompare. Plot containing the runtime of two-way comparison (A) and consensus TADs called on 4, 8, 12, and 16 replicates (B). Each point represent the runtime for a specific chromosome. X-axis - Chromosome, Y-axis - Runtime in seconds. Hi-C data from (Rao et al. 2014), chr 1-22, 10kb, 25kb, 50kb, and 100kb resolution.

Supplementary Table Legends

Supplementary Table 1. Contact matrix data sources. The source of all contact matrices, experimental and simulated, used in this paper are provided. Experimental data are seperated based on study and cell line.

Supplementary Table 2. Genomic annotation data sources. The sources, with download links, for all genomic annotation used in this paper are included.

Supplementary Table 3. Summary of differential boundary types across tissues and cell-lines. The percentage of each type of differential boundary for all tissue-tissue and cell line-cell line comparisons is reported. Results are aggregated over all chromosomes. Hi-C data from Schmitt et al. (Schmitt et al. 2016), 40kb resolution, chr 1-22.

Supplementary Table 4. Gene ontology enrichment for differential boundary types. Differential boundaries were identified between the neural progenitor cells (NPC) and mesenchymal stem cells (MSC) (Schmitt et al. 2016). Pathway analysis was performed using `rGREAT` (Methods) and results are seperated by ontology. Boundaries with an FDR adjusted p-value of <0.3 are shown. 40kb resolution, chr1-22.

Supplementary Table 5. Gene ontology enrichment between the first and last time point in auxin-treated data . Differential boundaries were identified between the first and last time point of auxin-treated data (Rao et al., n.d.). Pathway analysis was performed using `rGREAT` (Methods) and results are seperated by ontology. Boundaries with an FDR adjusted p-value of <0.3 are shown. 50kb resolution, chr1-22.

Supplementary Table 6. Enrichment across different temporal boundary types. Temporal boundary types were identified across four time points in auxin-treated data (Rao et al., n.d.). Results are shown for four types of temporal TAD (Early Appearing, Late Appearing, Highly Common, Dynamic). Permutation p-values, along with enrichment or depletion designations, are reported. HCT-116 cell line, 40kb resolution, chr 1-22.

Supplementary Table 7. Gene ontology enrichment for different temporal boundary types. Temporal boundary types were identified across four time points in auxin-treated data (Rao et al., n.d.). For each temporal boundary type, pathway analysis was performed using `rGREAT` (Methods) and results are seperated by ontology. Boundaries with an FDR adjusted p-value of <0.3 are shown. HCT-116 cell line, 50kb resolution, chr1-22.

Supplementary Table 8. Enrichment across different consensus scores. Consensus scores were called across 17 contact matrices representing 7 different cell lines. Results were dichotomized into three groups (<2, 2-4, >4) based on consensus boundary scores.

Permutation p-values, along with enrichment or depletion designations, are reported. Hi-C data from Schmitt et al. (Schmitt et al. 2016), 40kb resolution, chr 1-22.

References

Chen, Fengling, Guipeng Li, Michael Q Zhang, and Yang Chen. 2018. "HiCDB: A Sensitive and Robust Method for Detecting Contact Domain Boundaries." *Nucleic Acids Research*, September. https://doi.org/10.1093/nar/gky789.

Crane, Emily, Qian Bian, Rachel Patton McCord, Bryan R Lajoie, Bayly S Wheeler, Edward J Ralston, Satoru Uzawa, Job Dekker, and Barbara J Meyer. 2015. "Condensin-Driven Remodelling of X Chromosome Topology During Dosage Compensation." *Nature* 523 (7559): 240–4. https://doi.org/10.1038/nature14450.

Cresswell, Kellen G., John C. Stansfield, and Mikhail G. Dozmorov. 2019. "SpectralTAD: An R Package for Defining a Hierarchy of Topologically Associated Domains Using Spectral Clustering," February. https://doi.org/10.1101/549170.

Dali, Rola, and Mathieu Blanchette. 2017. "A Critical Assessment of Topologically Associating Domain Prediction Tools." *Nucleic Acids Res* 45 (6): 2994–3005. https://doi.org/10.1093/nar/gkx145.

Dixon, Jesse R, Siddarth Selvaraj, Feng Yue, Audrey Kim, Yan Li, Yin Shen, Ming Hu, Jun S Liu, and Bing Ren. 2012. "Topological Domains in Mammalian Genomes Identified by Analysis of Chromatin Interactions." *Nature* 485 (7398): 376–80. https://doi.org/10.1038/nature11082.

Dong, Qianli, Ning Li, Xiaochong Li, Zan Yuan, Dejian Xie, Xiaofei Wang, Jianing Li, et al. 2018. "Genome-Wide Hi-c Analysis Reveals Extensive Hierarchical Chromatin Interactions in Rice." *Plant J* 94 (6): 1141–56. https://doi.org/10.1111/tpj.13925.

Filippova, Darya, Rob Patro, Geet Duggal, and Carl Kingsford. 2014. "Identification of Alternative Topological Domains in Chromatin." *Algorithms for Molecular Biology* 9 (1): 14. https://doi.org/10.1186/1748-7188-9-14.

Forcato, Mattia, Chiara Nicoletti, Koustav Pal, Carmen Maria Livi, Francesco Ferrari, and Silvio Bicciato. 2017. "Comparison of Computational Methods for Hi-c Data Analysis." *Nat Methods* 14 (7): 679–85. https://doi.org/10.1038/nmeth.4325.

Fraser, James, Carmelo Ferrai, Andrea M Chiariello, Markus Schueler, Tiago Rito, Giovanni Laudanno, Mariano Barbieri, et al. 2015. "Hierarchical Folding and Reorganization of Chromosomes Are Linked to Transcriptional Changes in Cellular Differentiation." *Mol Syst Biol* 11 (12): 852.

Gibcus, Johan H, and Job Dekker. 2013. "The Hierarchy of the 3D Genome." *Mol Cell* 49 (5): 773–82. https://doi.org/10.1016/j.molcel.2013.02.011.

Rao, Suhas S. P., Su-Chen Huang, Brian Glenn St Hilaire, Jesse M. Engreitz, Elizabeth M. Perez, Kyong-Rim Kieffer-Kwon, Adrian L. Sanborn, et al. n.d. "Cohesin Loss Eliminates

All Loop Domains.” *Cell* 171 (2): 305–320.e24. https://doi.org/10.1016/j.cell.2017.09.026.

Rao, Suhas S.P., Miriam H. Huntley, Neva C. Durand, Elena K. Stamenova, Ivan D. Bochkov, James T. Robinson, Adrian L. Sanborn, et al. 2014. “A 3D Map of the Human Genome at Kilobase Resolution Reveals Principles of Chromatin Looping.” *Cell* 159 (7): 1665–80. https://doi.org/10.1016/j.cell.2014.11.021.

Sauerwald, Natalie, and Carl Kingsford. 2018. “Quantifying the Similarity of Topological Domains Across Normal and Cancer Human Cell Types.” *Bioinformatics* 34 (13): i475–i483. https://doi.org/10.1093/bioinformatics/bty265.

Sauerwald, Natalie, Akshat Singhal, and Carl Kingsford. 2018. “Analysis of the Structural Variability of Topologically Associated Domains as Revealed by Hi-c:” December. https://doi.org/10.1101/498972.

Schmitt, Anthony D, Ming Hu, Inkyung Jung, Zheng Xu, Yunjiang Qiu, Catherine L Tan, Yun Li, et al. 2016. “A Compendium of Chromatin Contact Maps Reveals Spatially Active Regions in the Human Genome.” *Cell Rep* 17 (8): 2042–59. https://doi.org/10.1016/j.celrep.2016.10.061.

Weinreb, Caleb, and Benjamin J Raphael. 2016. “Identification of Hierarchical Chromatin Domains.” *Bioinformatics* 32 (11): 1601–9. https://doi.org/10.1093/bioinformatics/btv485.

Zaborowski, Rafal, and Bartek Wilczynski. 2016. “DiffTAD: Detecting Differential Contact Frequency in Topologically Associating Domains Hi-c Experiments Between Conditions,” December. https://doi.org/10.1101/093625.

Zufferey, Marie, Daniele Tavernari, Elisa Oricchio, and Giovanni Ciriello. 2018. “Comparison of Computational Methods for the Identification of Topologically Associating Domains.” *Genome Biology* 19 (1). https://doi.org/10.1186/s13059-018-1596-9.
