# Supplementary figures and images for: TADCompare: An R Package for Differential and Temporal Analysis of Topologically Associated Domains
Source: Front Genet. 2020 Mar 10;11:158. doi: 10.3389/fgene.2020.00158 (PMC7076128; doi:10.3389/fgene.2020.00158)

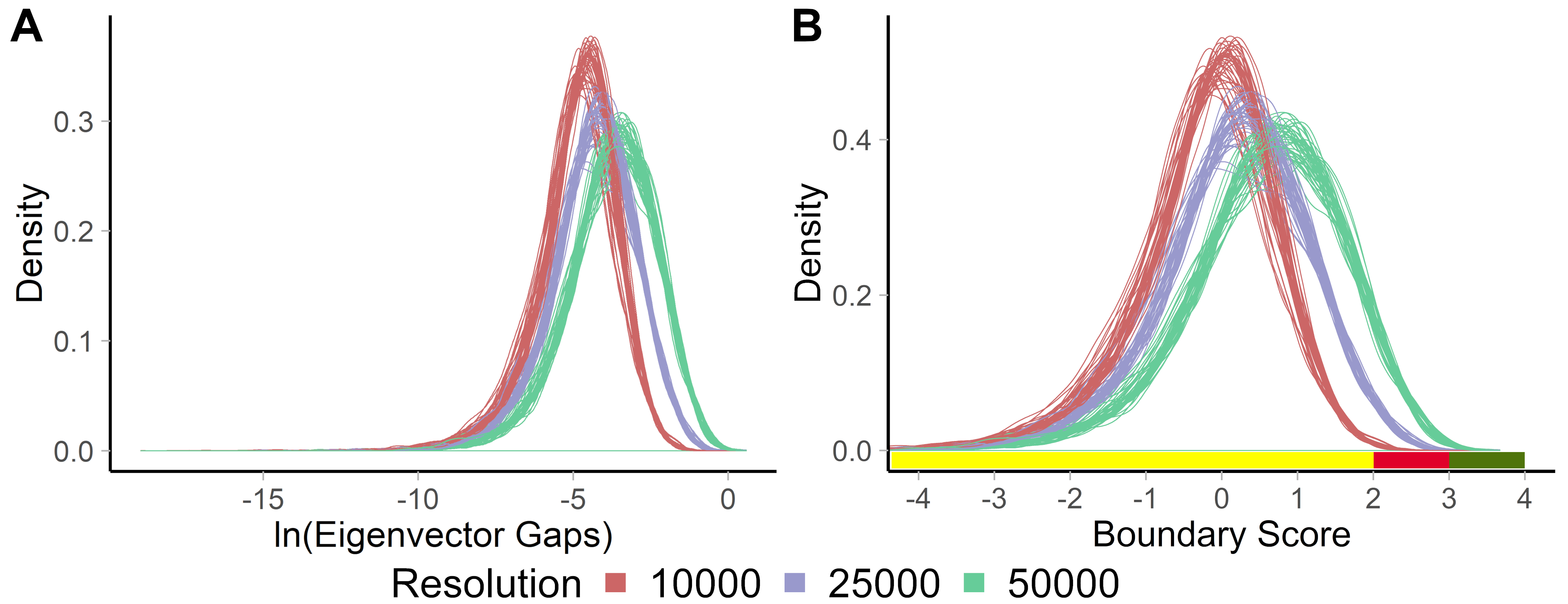

Supplement: Supplementary Figure 1 — Log-normal distribution of eigenvector gaps converted to boundary Z-scores. Eigenvector gaps were calculated for contact matrices across three resolutions [10, 25, and 50 kb, Hi-C data from Rao et al. (2014), GM12878 cell line, chr 1–22]. Density plots are shown for the (A) Natural log of the eigenvector gaps and (B) Boundary scores derived from the same data, separated by resolution. Regions of non-TADs are highlighted by a yellow bar, moderate strength boundaries (2 < boundary score cutoff <3) are highlighted by a red bar, and strong boundaries (cutoff > 3) are shown using a green bar. We see a slightly right-skewed distribution due to the filtering of gaps for plotting purposes. [file Image_1.TIFF]

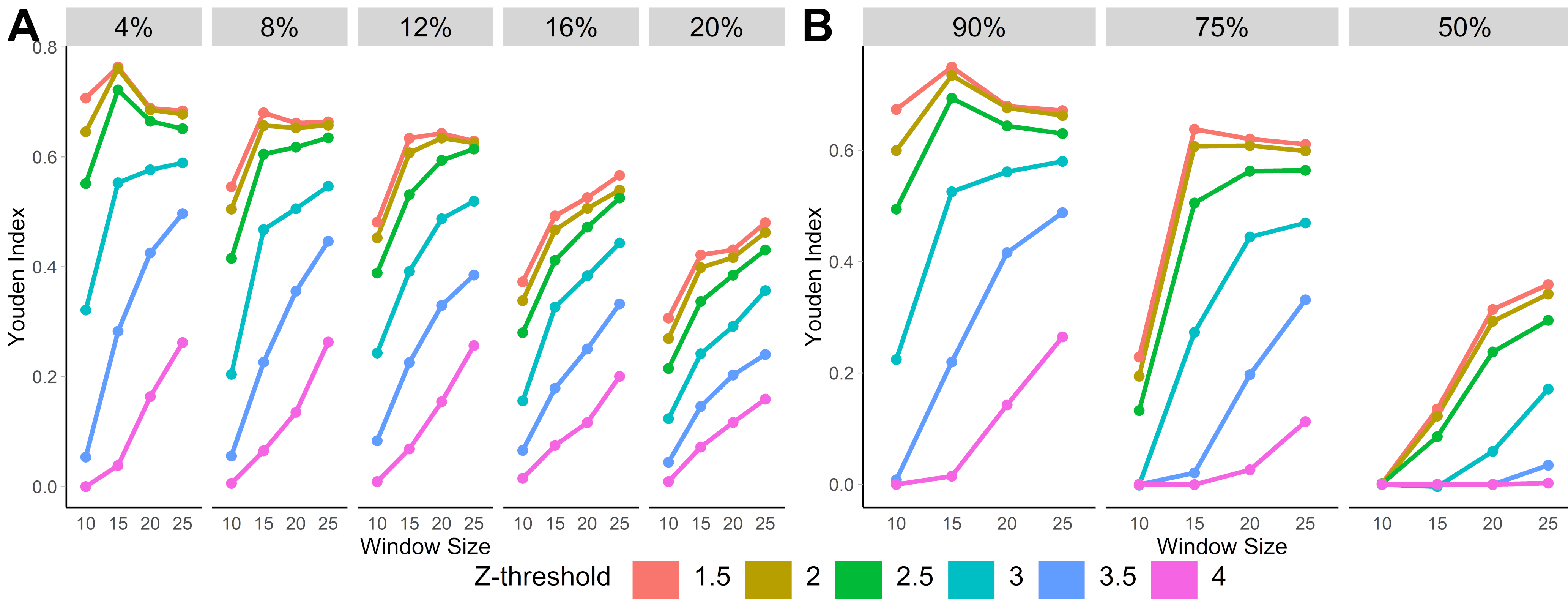

Supplement: Supplementary Figure 2 — Window size of 15 units of Hi-C data resolution and boundary score cutoff of 2 yields consistent boundary detection. Differential boundaries were compared between two simulated data sets with window size sizes ranging from 10 to 25, and boundary score cutoff ranging from 1.5 to 4. Youden index (balanced sensitivity and specificity metric) was calculated for each combination and plotted to show agreement with ground-truth annotations. Results are shown for noise-injected matrices (A) and sparsity-injected matrices (B). [file Image_2.TIFF]

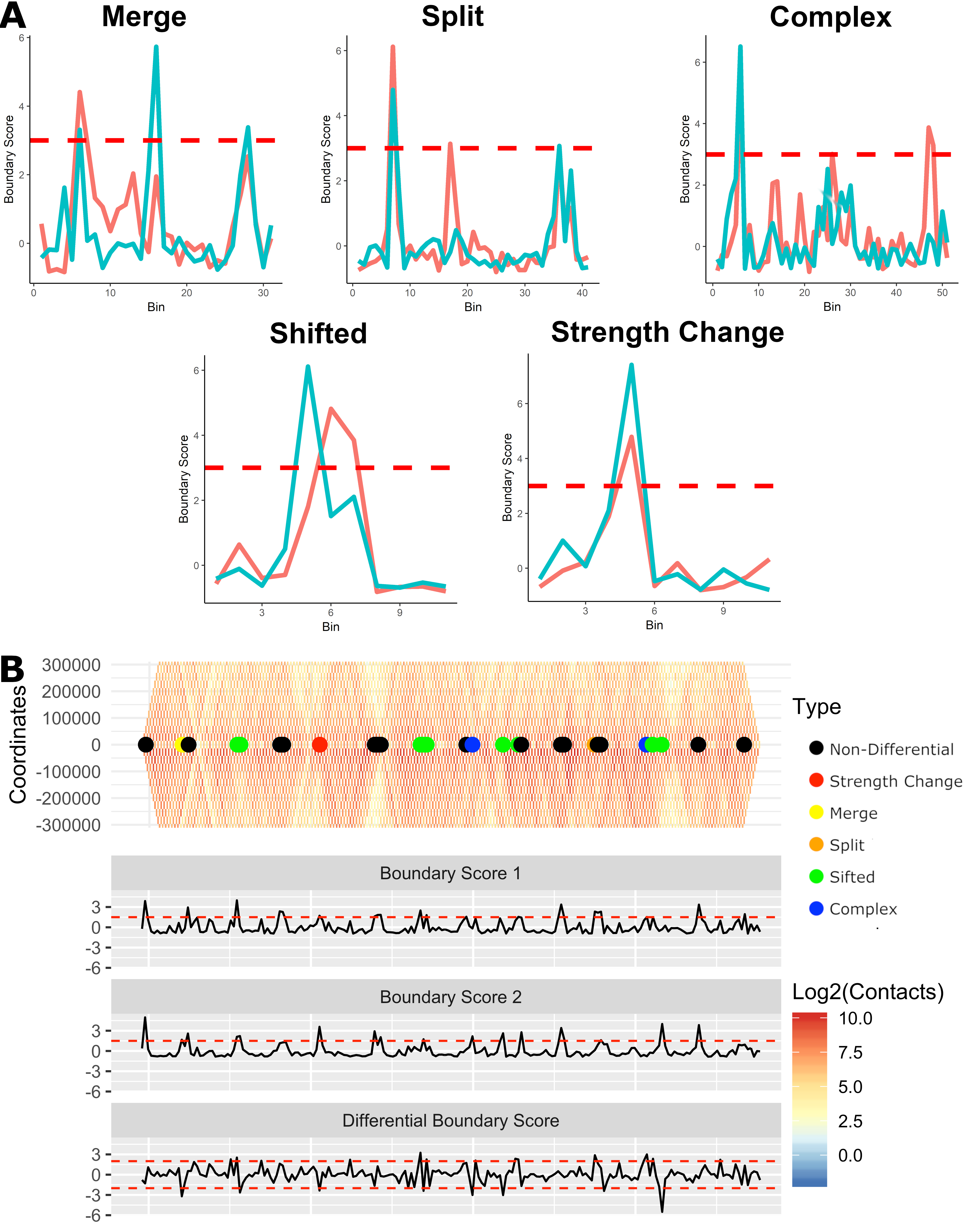

Supplement: Supplementary Figure 3 — Visualization of different types of boundary score patterns. (A) Patterns of raw boundary scores are shown for five different types of differential boundaries (Merge, split, complex, shifted, and strength change). The red horizontal line corresponds to the user-adjustable cutoff for a boundary. Human neural progenitor cells (NPCs), chr22, most representative examples are shown. (B) TADCompare::DiffPlot differential boundary visualization between NPCs and mesenchymal stem cells (MSC), chr4:10500000–18600000. 40 kb resolution data from Schmitt et al. (2016). [file Image_3.TIFF]

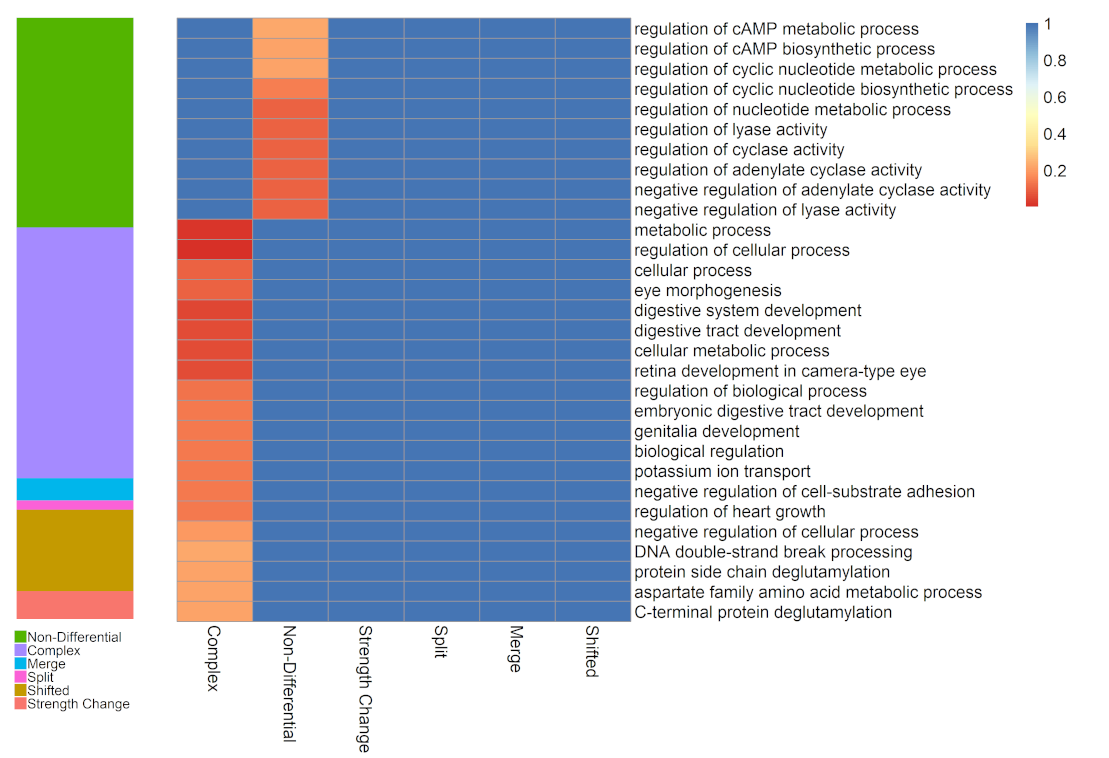

Supplement: Supplementary Figure 4 — Heatmap of gene ontology enrichment at the first and last time point in auxin-treated data. Differential boundary identification was performed on auxin-treated data at the time of application (first time point) and complete withdrawal (last time point) [HCT-116 cell line, chr 1–22, 40 kb resolution (Schmitt et al., 2016)]. A barplot of the proportion of each differential boundary type and FDR-adjusted hypergeometric p-values obtained from gene ontology enrichment analysis using rGREAT (see Methods) are shown. The top 30 pathways, in terms of average enrichment, are shown and clustered using the Ward method. [file Image_4.TIFF]

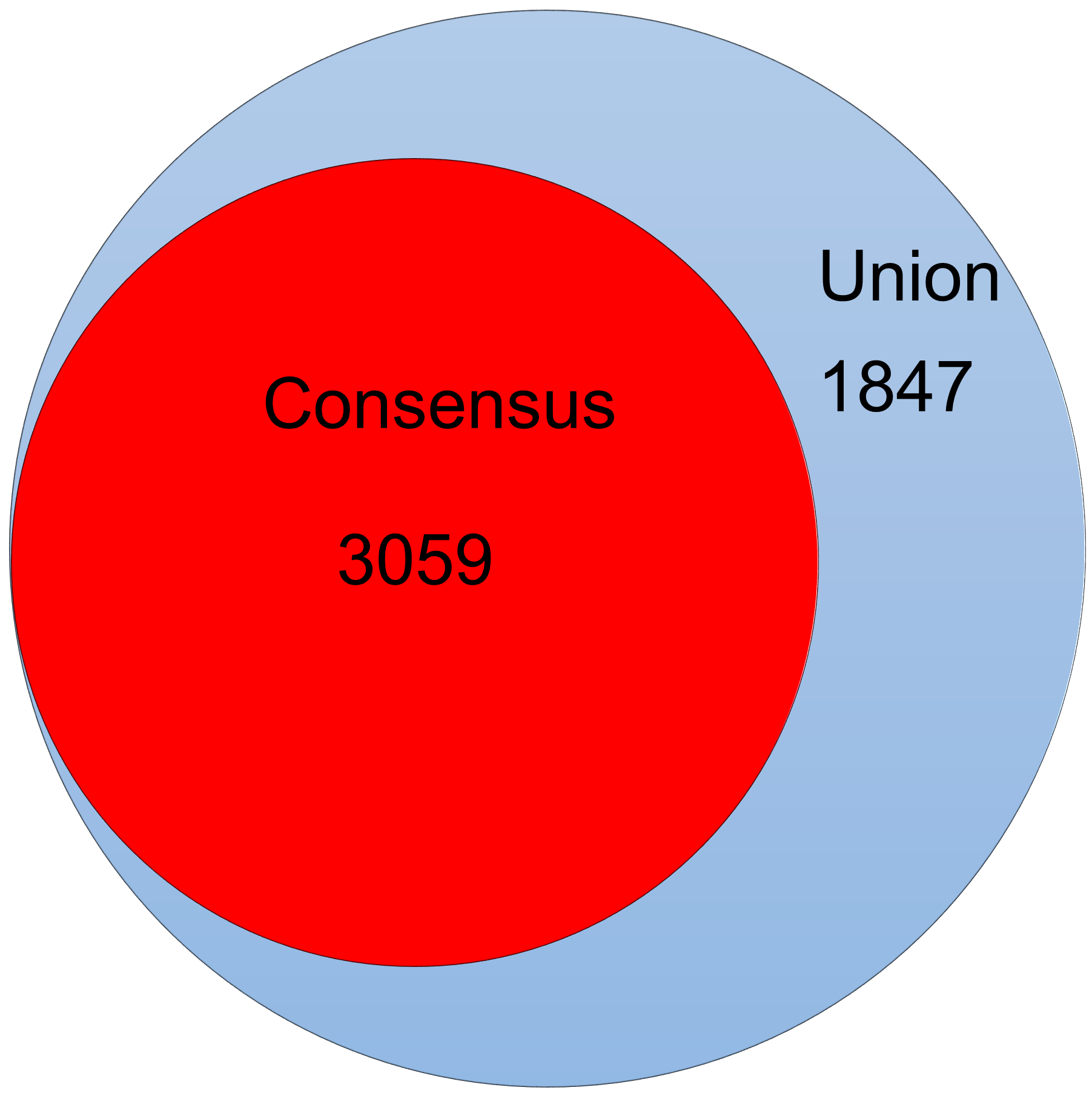

Supplement: Supplementary Figure 5 — Venn diagram of union and consensus boundary counts. Consensus and union boundaries were called across four different cell lines (hesc, mesynchymal, npc, trophectoderm), and the number of union and consensus boundaries was recorded. The Venn diagram shows the complete overlap of consensus boundaries within union boundaries (40 kb resolution, data from Schmitt et al., 2016). [file Image_5.TIFF]

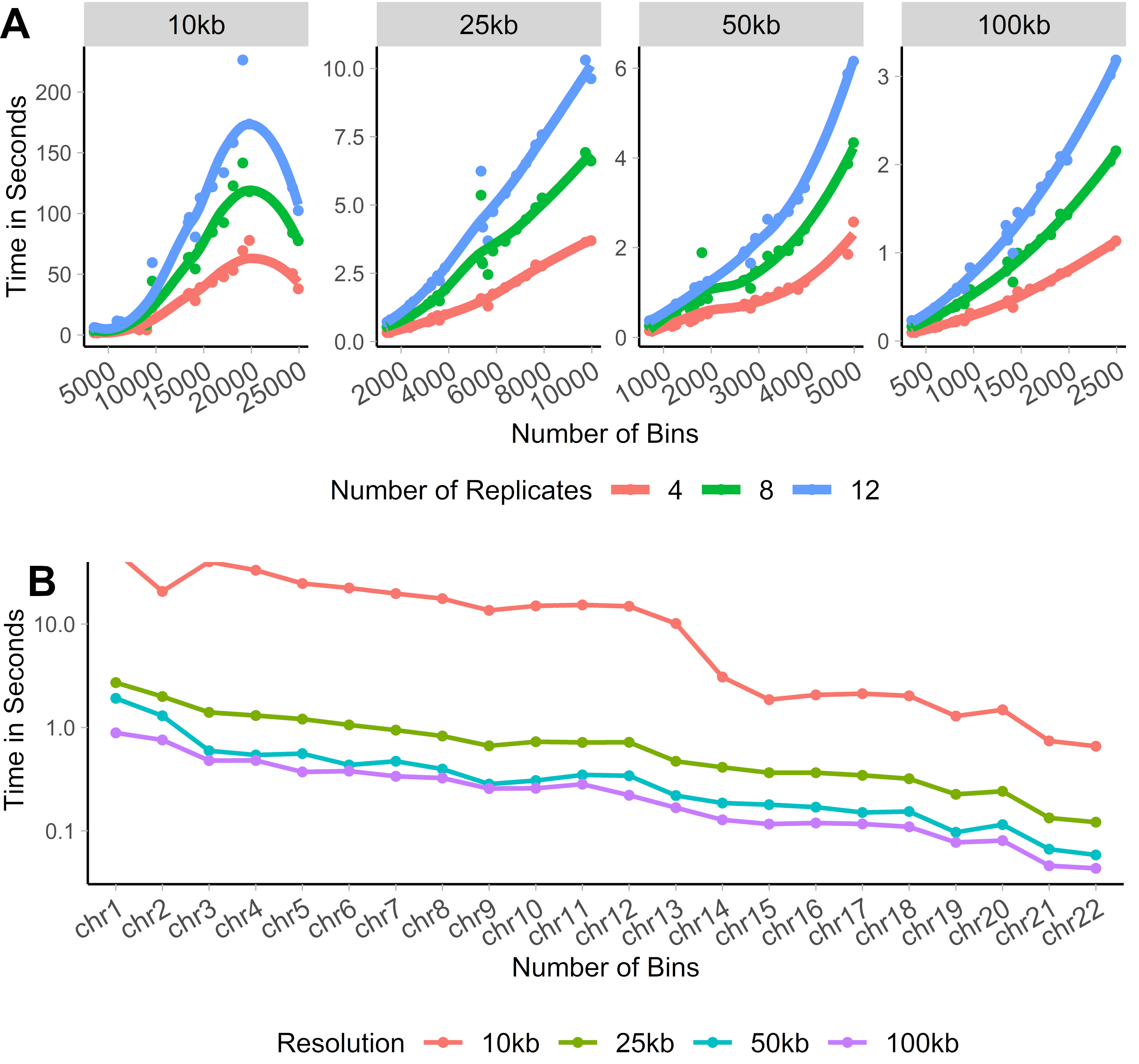

Supplement: Supplementary Figure 6 — Runtime of TADCompare. Plot containing the runtime of two-way comparison (A) and consensus boundaries called on 4, 8, 12, and 16 replicates (B). Each point represents the runtime for a specific chromosome. X-axis—chromosome, Y-axis—runtime in seconds. Hi-C data from Rao et al. (2014), chr 1–22, 10, 25, 50, and 100 kb resolution. [file Image_6.TIFF]
